# Modulation of immunosuppressive cells and noncoding RNAs as immunotherapy in osteosarcoma

**DOI:** 10.3389/fimmu.2022.1025532

**Published:** 2022-11-15

**Authors:** Yidan Xia, Dongxu Wang, Yuting Piao, Minqi Chen, Duo Wang, Ziping Jiang, Bin Liu

**Affiliations:** ^1^ Department of Hand and Foot Surgery, The First Hospital of Jilin University, Changchun, China; ^2^ Laboratory Animal Center, College of Animal Science, Jilin University, Changchun, China

**Keywords:** osteosarcoma, noncoding RNA, immunosuppressive cells, TME, immunotherapy, metabolic heterogeneity

## Abstract

The most common bone cancer is osteosarcoma (OS), which mostly affects children and teenagers. Early surgical resection combined with chemotherapy significantly improves the prognosis of patients with OS. Existing chemotherapies have poor efficacy in individuals with distant metastases or inoperable resection, and these patients may respond better to novel immunotherapies. Immune escape, which is mediated by immunosuppressive cells in the tumour microenvironment (TME), is a major cause of poor OS prognosis and a primary target of immunotherapy. Myeloid-derived suppressor cells, regulatory T cells, and tumour-associated macrophages are the main immunosuppressor cells, which can regulate tumorigenesis and growth on a variety of levels through the interaction in the TME. The proliferation, migration, invasion, and epithelial–mesenchymal transition of OS cells can all be impacted by the expression of non-coding RNAs (ncRNAs), which can also influence how immunosuppressive cells work and support immune suppression in TME. Interferon, checkpoint inhibitors, cancer vaccines, and engineered chimeric antigen receptor (CAR-T) T cells for OS have all been developed using information from studies on the metabolic properties of immunosuppressive cells in TME and ncRNAs in OS cells. This review summarizes the regulatory effect of ncRNAs on OS cells as well as the metabolic heterogeneity of immunosuppressive cells in the context of OS immunotherapies.

## Introduction

Osteosarcoma (OS) is a malignant mesenchymal tumour that most commonly affects children and adolescents and has a high rate of metastasis and mortality (1). OS primarily affects the epiphysis of the long bones in the extremities, with lung metastases occurring most frequently (2). Current treatments for OS include surgical resection and adjuvant chemotherapy, which typically result in a survival rate of less than 5 years for patients with distant metastases (3). Metastatic OS has been successfully treated with immunotherapy, and the mechanisms underlying this success are related to the heterogeneity of immunosuppressive cells in metastatic tumours and the interaction of stromal and immunosuppressive cells in the tumour microenvironment (TME) (4).

The TME in OS is complex and diverse and plays a critical role in tumorigenesis and development. The TME consists of stromal cells and other key factors, including cancer-associated fibroblasts (CAFs), immune cells, extracellular matrix, and vasculature (5). To promote the occurrence and development of tumour cells, stromal cells secrete cytokines, growth factors, and chemokines (6). Immune cells such as lymphocytes and natural killer cells can effectively control tumour invasion, which can be suppressed by immunosuppressive cells such as myeloid-derived suppressor cells (MDSCs), regulatory T cells (Tregs), and tumour-associated macrophages (TAMs) (7). Immunosuppressive cells and stromal cells in the TME mutually promote the growth and maturation of OS cells (8).

The proliferation, angiogenesis, and apoptosis of OS cells are closely related to noncoding RNAs (ncRNAs), including microRNAs (miRNAs), long noncoding RNAs (lncRNAs), and circular RNAs (circRNAs) (9, 10). miRNAs can regulate the proliferation and apoptosis of OS cells *via* their aberrant expression (11). Overexpression of mir-542-5p can enhance proliferation, but miRNA-1236-3p can decrease proliferation and promote apoptosis in OS cells (12, 13). lncRNAs can enhance OS progression, such as SNHG3, whose overexpression can speed up the migration and invasion of OS cells (14). circRNAs function as a miRNA sponge, regulating transcriptional or post-transcriptional gene expression and contributing to the control of OS incidence and development (15). lncRNA and circRNA can regulate the biological activity of OS cells by forming miRNA sponge, which act as competitive endogenous RNA (ceRNA) (10, 16). Studies on the metabolic properties of immunosuppressive cells and ncRNAs in OS cells promote the use of immunotherapy in the treatment of OS, including interferon treatments, checkpoint inhibitors, cancer vaccines, and engineered chimeric antigen receptor T (CAR-T) cells (4, 17). Among these, CAR-T cell treatment offers a significant advancement in T-cell-based immunotherapy and is predicted to be a game changer in OS immunotherapies (18). We summarize the metabolic properties of immunosuppressive cells in the TME and functional ncRNAs in OS in this paper. The targets, efficacy, and drug resistance of several recently developed immunotherapies are compared.

## Noncoding RNAs in osteosarcoma

The pathophysiology of OS is related to aberrant oncogene activation and tumour suppressor gene inactivation induced by somatic mutations and epigenetic processes (19). Recent studies have increasingly focused on the dysregulation of ncRNAs, including miRNAs, lncRNAs, and circRNAs (9, 20).

### MicroRNAs

**Figure 1:**
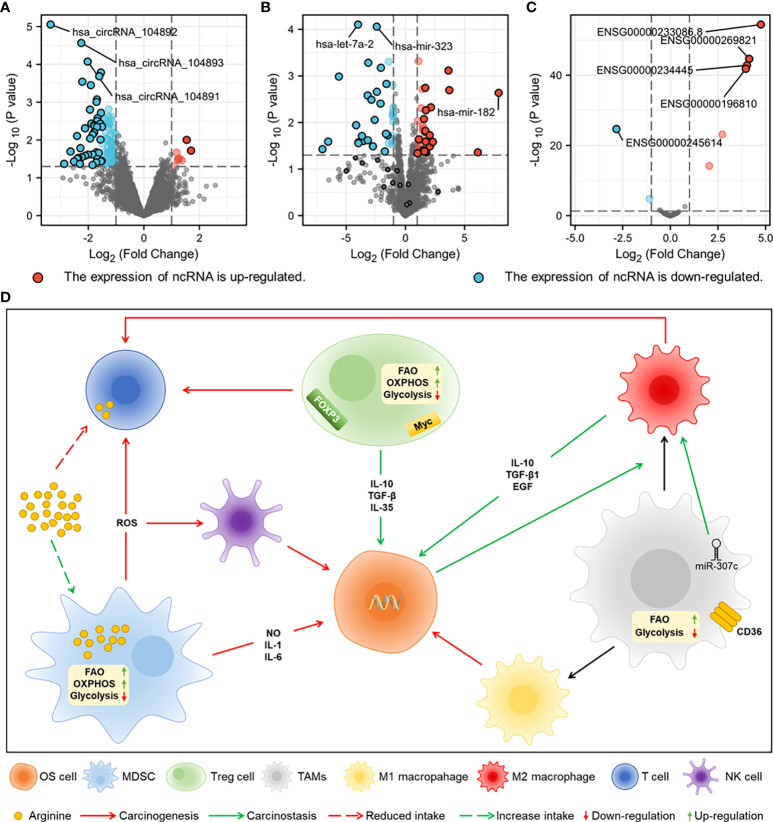
The sequencing results of ncRNAs from normal tissues and OS tissues of human are downloaded from GEO datasets of National Center for Biotechnology Information (NCBI) for difference analysis. **(A)** Differential expression analysis of miRNAs (GSE70367). **(B)** Differential expression analysis of lncRNAs (GES156344). **(C)** Differential expression analysis of circRNAs (GSE96964). **(D)** Metabolic characteristics of MDSC, TAMs and Treg cells in TME and crosstalk of them with NK cells, T cells and OS cells. MDSC promotes the growth of OS cells by secreting inflammatory factors, damages T cells and NK cells by secreting ROS, and competitively consumes arginine with T cells. Treg cells inhibit T cell function by expressing FOXP3 and myc. CD36 receptor and miR-307c in TAMs promote M2 polarization, and FAO is the main energy metabolism.

miRNAs regulate cell proliferation, differentiation, apoptosis, and development by binding to the 3’ untranslated region (3’-UTR) of target mRNAs and are able to degrade or induce translational silencing in OS cells (21). miR-223-3p has been shown in studies to limit cadherin-6 expression by directly binding to the 3’-UTR of cadherin-6 and to inhibit the invasion, migration, growth, and proliferation of OS cells (22). The expression of miR-18b-5p, which is mediated by HIF-1α, is substantially increased in OS and is associated with a poor prognosis (23). In addition, miR-18b-5p promotes the incidence and development of OS by inhibiting the expression of the tumour suppressor gene PHF2 (23). miRNA-98-5p is under-expressed in OS and inhibits cell cycle progression and migration potential by down-regulating CDC25A, thereby inducing OS apoptosis (24). Overexpression of miRNA-1236-3p in HOS cells reduces proliferation, stops the cell cycle in the G0/G1 phase, and promotes apoptosis (13). A differential analysis of miRNA expression in OS (**Figure 1A**) shows that the expression of let-7A-2 and miR-323 is decreased, whereas the expression of miR-182 is increased, suggesting that miR-182 could be a possible therapeutic target in OS. The detailed information of differentially expressed ncRNAs in A-C is presented in **Table S1**.

### Long noncoding RNAs

The expression of lncRNA MELTF-AS1 is significantly increased in OS and promotes OS metastasis by upregulating the expression of MMP14 (25). lncRNA ODRUL can act as a competitive endogenous RNA (ceRNA) sponge of miR-3182 and promotes the proliferation, migration, invasion, and tumour growth of OS by upregulating the expression of matrix metalloproteinase (MMP) II (26). The oncogenic effects of LncRNA CBR3-AS1 are executed by regulating the network of the miR-140-5p/DDX54-NucKS1-mTOR signalling pathway, which encourages stemness and epithelial–mesenchymal transition (EMT) of OS (27). The overexpression of lncRNA EBLN3P promotes the progression of OS cells, which is indicative of the stimulating effects of EBLN3P (28). In OS cells, the expression of the lncRNAs ENSG00000233086.8 and ENSG00000269821 is much higher (**Figure 1B**). By examining the molecular pathways and regulatory mechanisms further, one may be able to control the development of OS.

### Circular RNAs

circECE1 is highly expressed in OS tissues and cells, and its association with c-Myc promotes tumour proliferation and metastasis by boosting glucose metabolism in OS cells to prevent speckle-type POZ-mediated ubiquitination and degradation of c-Myc (29). C-Myc-targeting checkpoint inhibitors have been demonstrated to impede OS development *via* modulating the production of ncRNAs (30). Studies have shown that knockdown of circRNA circ_001422 significantly inhibits the proliferation and metastasis of OS cells and promotes apoptosis. Regulating the miR-195-5p/FGF2/PI3K/AKT axis produces the opposite impact of overexpression (31). circMYO10 has been confirmed as a promoter of OS progression by regulating the miR-370-3p/RUVBL1 axis and chromatin remodelling, consequently boosting the transcriptional activity of the β-catenin/LEF1 complex (32). The number of circRNAs with decreased expression was much greater than those with enhanced expression (**Figure 1C**), a finding that could be leveraged to design targeted therapies once the regulatory mechanisms of these circRNAs have been elucidated.

Recent research has increasingly focused on the impacts and mechanisms of microRNAs, whereas research into lncRNAs and circRNAs is still in its infancy (**Table 1**) (102). More research points to the importance of noncoding RNAs in OS, both in terms of diagnosis and treatment (9). An alternative mechanism for OS chemotherapeutic resistance has been proposed through the construction of ceRNA networks, in which noncoding RNAs bind to mRNAs (103). Differential expression of noncoding RNAs and the formation of ceRNA networks may lead to the development of more effective treatment techniques and the ability to overcome drug resistance in OS (**Figure S1**).

**Table 1:** Regulation of miRNAs, lncRNAs and circRNAs in OS cells.

Non-coding RNA	Expression	Function	Ref
miR-873	upregulate	Related to tumor size, clinical stage and distant metastasis in OS.	(33)
miR-23b-3p	upregulate	Inhibit OS cell proliferation.	(34)
miR-367	upregulate	Inhibit the proliferation, migration and invasion of OS cells.	(35)
miR-21	upregulate	Play a main role in proliferation, migration, invasion and apoptosis.	(36)
miR-107	upregulate	Promoted U_2_OS cell viability, migration, and invasion whereas inhibit apoptosis.	(37)
miR-590-3p	downregulate	Inhibit proliferation and metastasis in OS cells.	(38)
miR-520a-3p	downregulate	Tumor suppressor.	(39)
miR-491	downregulate	Stimulate OS cell lung metastasis and suppresses CDDP-induced tumor growth inhibition and apoptosis.	(40)
miR-449a	downregulate	Decrease cyclin A2 levels and inhibit proliferation rate, migratory potential, and colony-forming ability of OS cells.	(41)
miR-432-5p	downregulate	Regulate SA and IA by targeting PDGFB genes.	(42)
miR-425-5p	downregulate	Suppress OS cell proliferation, invasion and migration *in vitro*.	(43)
miR-424	downregulate	Decrease cyclin A2 levels and inhibited proliferation rate, migratory potential, and colony-forming ability of OS cells.	(41)
miR-377	downregulate	Inhibit tumor growth and reduce tumor size.	(44)
miR-363-3p	downregulate	Inhibit the proliferation, migration, and invasion of U_2_OS and MG63 cells.	(45)
miR-342-3p	downregulate	Inhibit the proliferation, migration, and invasion of OS cells.	(46)
miR-26a	downregulate	Suppress the malignant behaviors of OS cells.	(47)
miR-223-3p	downregulate	Inhibit cell invasion, migration, growth, and proliferation.	(22)
miR211	downregulate	Increase the percentage of cell apoptosis, and suppress cell proliferation as well as cell migration/invasion.	(48)
miR-133b	downregulate	Attenuate cell proliferation and invasion.	(49)
miR-520b	downregulate	Inhibit cell proliferation, migration, and invasion.	(50)
miR-326	downregulate	Promote the proliferation and invasion of MG63 cells as well as the growth and metastasis in nude mice.	(51)
lncRNA MALAT1	upregulate	Promote OS cell viability, invasion and migration.	(52)
lncRNA TP73-AS1	upregulate	Suppress OS cells proliferation and invasion *in vitro* as well as tumor growth *in vivo*.	(53)
lncRNA HNF1A-AS1	upregulate	Inhibit cell proliferation and G1 /S transition, and suppress migration and invasion in OS cells.	(54)
lncRNA-BC050642	upregulate	Promote cell proliferation, induce colony formation and meanwhile inhibit cell apoptosis.	(55)
lncRNA ODRUL	upregulate	Inhibit OS cell proliferation, migration, invasion, and tumor growth *in vitro* and *vivo*.	(26)
lncRNA ITGB2-AS1	upregulate	Inhibit the proliferation and induce apoptosis of OS cells.	(56)
lncRNA ANRIL	upregulate	Associate with increased rates of metastases at diagnosis and death. A significant predictor of reduced overall survival rate.	(57)
lncRNA XIST	upregulate	Responsible for OS cell proliferation and invasion.	(58)
lncRNA TUG1	upregulate	Play an important role in the proliferation and metastasis of osteosarcoma.	(59)
lncRNA TUG1	upregulate	Regulate OS cell metastasis, angiogenesis, and proliferation *in vivo* and *vitro*.	(60)
lncRNA TNK2-AS1	upregulate	Inhibited proliferative, migratory, and invasive capacities while promoting apoptosis in OS cells.	(61)
lncRNA SNHG4	upregulate	Suppress cell viability and invasive potential.	(62)
lncRNA SNHG3	upregulate	Promote invasive and migratory potentials of OS cells.	(14)
lncRNA SNHG1	upregulate	Inhibit cell growth and metastasis of OS *in vitro* and *vivo*.	(63)
lncRNA SNHG16	upregulate	Contributes to the proliferation, migration and invasion of OS cells.	(64)
lncRNA OIP5-AS1	upregulate	Increased doxorubicin resistance of OS cells.	(65)
lncRNA OIP5-AS1	upregulate	Repress the proliferative ability and accelerated the apoptosis.	(66)
lncRNA MIR100HG	upregulate	Suppress cell proliferation, cell cycle progression while promote cell apoptosis.	(67)
lncRNA LINC01123	upregulate	Promote cell progression.	(68)
lncRNA LINC00324	upregulate	Accelerate the proliferation and migration of OS cells.	(69)
lncRNA KCNQ1OT1	upregulate	Facilitate proliferation and suppressed apoptosis of OS cells.	(70)
lncRNA JPX	upregulate	Elevate the cell viability and proliferation.	(71)
lncRNA HULC	upregulate	Promote OS cell proliferation, migration and invasion and induce cell apoptosis.	(72)
lncRNA HOXD-AS1	upregulate	Suppress cell proliferation, colony formation, migration, and invasion, and promote cell cycle arrest at G1 stage and apoptosis in OS cells.	(73)
lncRNA HOXD-AS1	upregulate	Inhibit the OS cells proliferation and induce G1/G0 phase arrest *in vitro*, and repress tumor cell growth *in vivo*.	(74)
lncRNA FOXD2-AS1	upregulate	Repress the malignant biological properties of OS cells *in vitro* and *vivo*, including proliferation, invasion, apoptosis and tumor growth.	(75)
lncRNA DLEU1	upregulate	Inhibit the cell proliferation, migration and invasion.	(76)
lncRNA DANCR	upregulate	Promote tumor growth and lung metastasis of OS.	(77)
lncRNA DANCR	upregulate	Increase OS cell proliferation, migration, and invasion.	(78)
lncRNA CCAT2	upregulate	Promote OS cell proliferation, cell cycle and invasion.	(79)
lncRNA CBR3-AS1	upregulate	Suppress OS cells proliferation, migration and invasion, and promote cells apoptosis.	(80)
lncRNA APTR	upregulate	Repress human OS cell proliferation, invasion and migration, and induce apoptosis.	(81)
lncRNA CAT104	upregulate	Inhibit OS-732 cell proliferation, migration, and invasion, but promote cell apoptosis.	(82)
lncRNA LINC01128	upregulate	Reduce the proliferation, migration and invasion of OS cells both.	(83)
lncRNA ZBTB7A	upregulate	Associate with OS metastasis.	(84)
lncRNA RSF1	upregulate	Suppress OS cells proliferation and invasion.	(85)
lncRNA PUM2	downregulate	Inhibit OS cells proliferation, migration, and stemness.	(86)
lncRNA XIST	downregulate	Inhibit the proliferation of OS cells.	(87)
lncRNA C2dat1	downregulate	Reduce cell viability, invasion, and migration, whereas increase cell apoptosis in OS-732 cells.	(88)
hsa_circ_0008934	upregulate	Reduce proliferation, enhanced apoptosis, block cell cycle progression, and impair migration and invasion capacities of SAOS_2_ cells.	(89)
hsa_circ_0007534	upregulate	Suppress OS cell growth.	(90)
circUSP34	upregulate	Promote the proliferation, migration, and invasion of OS *in vitro* and *vivo*.	(91)
circ-LRP6	upregulate	Inhibit the proliferation, migration and invasion of OS cells.	(92)
circUBAP2	upregulate	Inhibit OS cell apoptosis.	(93)
circTADA2A	upregulate	Increase malignant tumor behavior.	(94)
hsa_circ_0002137	upregulate	Suppress the progress of OS, including cell invasion, cell cycle and cell apoptosis.	(95)
circPVT1	upregulate	Suppress OS tumor growth and metastasis *in vivo*.	(96)
circECE1	upregulate	Suppress tumor proliferation and metastasis both *in vitro* and *vivo. *	(29)
circ_0078767	upregulate	Strengthen the proliferation, invasiveness, and migration of osteosarcoma cells.	(97)
circ_001621	upregulate	Promote OS proliferation and migration.	(98)
circ_001422	upregulate	Promote the proliferation and metastasis and inhibit the apoptosis of OS cells *in vivo* and *vitro.*	(31)
circ_0001721	upregulate	Facilitates cell progression in OS.	(99)
circ-0000658	downregulate	Promote cell cycle, proliferation, invasion and migration but inhibit the apoptosis of OS cells.	(100)
circ-0000190	downregulate	Exhibit an obvious reduction in tissues of OS patients.	(101)

## Immunosuppressive cells in osteosarcoma

### Myeloid-derived suppressor cells

Immature bone marrow cells (IMCs) differentiate into mature macrophages, dendritic cells, and granulocytes under physiological conditions and transform into immunosuppressive MDSCs when regulated by chemokines in the TME (104). MDSCs generate pro-inflammatory substances such as NO, IL-1, and IL-6, which expose OS cells to a persistently inflammatory environment and dramatically enhance the risk of DNA damage and tumour cell proliferation, which may contribute to the progression of OS (105, 106). Through the activation of the activator for transcription 3, miR-21 and IL-6 can synergistically enhance the development of MDSCs and influence treatment resistance (107). Reactive oxygen species (ROS) produced by oxidative stress can activate the NF-κB and Nrf2 pathways, allowing tumour cells to survive (108). MDSCs generate excessive ROS *via* NOX2 and suppress the antitumor effects of T cells and natural killer (NK) cells, hence mediating OS immune escape while maintaining oxidative balance *via* glycolysis upregulation (109, 110).

The TME alters the lipid metabolism of MDSCs to enhance the uptake of fatty acids and the activation of fatty acid oxidation (FAO), thereby improving the immunosuppressive activity of MDSCs and promoting tumour growth (110). In addition to LXR agonists, liver-X nuclear receptors (LXRs) regulate cholesterol and lipid metabolism *via* the transcription target Apolipoprotein E (111). LXR agonists have been demonstrated to play a role in MDSC depletion, which could be related to FAO inhibition in MDSCs (112). By increasing the activities of arginase-1, MDSCs compete with T cells for the consumption of arginine, which leads to T cell dysfunction (113). L-arginine supplementation may improve the anticancer impact of cyclophosphamide (CP) and minimize T cell dysfunction caused by increased MDSCs generated by CP (114).

### Tumour-associated macrophages

TAMs are the primary immune cells in the TME, which are usually produced from bone marrow monocytes, and the presence of TAMs is indicative of a poor prognosis in OS patients (115, 116). TAMs, *via* stimulating the COX-2/STAT3 axis and causing epithelial– mesenchymal transition, can increase OS pulmonary metastasis (117). C–C motif chemokine ligand 18 secreted by TAMs has been shown to promote the proliferation and metastasis of OS cells *via* the EP300/UCA1/Wnt/β-catenin pathway, which significantly reduces the survival rate of OS patients (118). Studies have demonstrated that miR-363 inhibitors can promote the migration of TAMs after transfection of OS cells (119).

TAMs can be divided into classically activated macrophages (M1), with antitumor activity, and selectively activated macrophages (M2), with tumour-promoting activity, both of which can coexist in the TME (120). It has been found that M2 can promote the deterioration of OS cells through the SOCS3/JAK2/STAT3 axis, and OS cells can enhance the M2 polarisation of TAMs (121). LncRNA RP11-361F15.2 enhances M2 polarisation mediated by cytoplasmic polyadenylate element binding protein 4 through miR-30c-5p and further promotes the occurrence of OS (122).

TAMs substitute glycolysis with FAO as a source of energy by expressing a high amount of the scavenger receptor CD36, which enhances lipid accumulation and reprograms TAMs into M2 types (123). S100A4 has been reported in mice to upregulate FAO and mediate TAM polarization to M2, as well as to have carcinogenic activity (124).

### Treg cells

Extensive Treg cell infiltration into tumour tissues is often associated with a poor prognosis, whereas their removal enhances antitumor immune responses (125). FOXP3+ expression in Treg cells has been shown to predict the prognosis of osteosarcoma *in vivo* and *in vitro* and could potentially be used as a diagnostic marker in clinical practice (126–128).

Glycolysis and oxidative phosphorylation, which are essential for Treg cell metabolism, require FAO (129). Treg cells in tumours, in contrast to normal tissues, have considerably decreased glucose uptake and are dysfunctional in a high-glucose environment (130). P13K inhibitors can reduce the immunosuppressive effects of Treg cells by upregulating glycolysis and reducing FOPX3 expression (131). miR-34a targets the 3’ UTR to inhibit the expression of FOXP3, which is controlled by the NF-κB pathway and downregulated by IL-6 and TNF-α (132). It has been demonstrated that the transcriptional regulator c-Myc influences oxidative phosphorylation in Tregs *via* regulating mitochondrial activity, hence limiting accumulation and functional activation (133). Targeting c-Myc and associated signalling pathways as a means of treating OS has drawn a lot of interest (29, 134).

Immunosuppressive cells can regulate the occurrence and development of OS through crosstalk with stromal cells in the TME (**Figure 1D**), which are regulated by ncRNAs in OS cells, according to the studies on the metabolic heterogeneity of immunosuppressive cells and the regulatory mechanisms of ncRNAs.

## Immunotherapy in osteosarcoma

### Interferon therapy

Interferon (IFN) is a cytokine that white blood cells generally secrete during infections (135). Due to its effects as an agonist of antitumor activity in adaptive and innate immune cells, it leads to the establishment of antiproliferative and antiangiogenic activity in osteosarcoma and antagonizes inhibitory immune subsets (135, 136). IFN-γ induces PKR-dependent autophagy in OS cells through signal transduction and activation of transcription 1, phosphatidylinositol 3-kinase, and mitogen-activated protein kinase-dependent pathways (137). miR-142-5p enhances the transcription of IFN-γ by downregulating the expression of interaction protein domain 2 (138). miR-31 reduces interferon-γ production, thereby attenuating Th1 response (139). The efficiency of IFN therapy could be increased by modulating the aberrant expression of ncRNAs, which has a good synergy for drug development in the treatment of OS.

### Checkpoint inhibitors

#### PD-1

In the tumour microenvironment of OS, PD-L1 on tumour cells interacts with PD-1 on T cells to inhibit T cell functional signalling, preventing the immune system from targeting tumour cells (140, 141). The antitumor activity of PD-1 can be aided by an SGLT2 inhibitor, and the synergistic effect stimulates the infiltration of CD4^+^ and CD8^+^T lymphocytes into the OS tumour microenvironment (142). miR-140 was found to directly regulate the expression of PD-L1 by binding to its 3’-UTR, suggesting that it could be exploited as a new therapeutic drug targeting checkpoint inhibitors in OS (143). PBMC-loaded vMyx-hTNF may synergistically interact with the immune checkpoint inhibitor anti-PD-1, which has been reported in a mouse model of lung metastatic osteosarcoma (144).

#### C-Myc inhibitors

The ubiquitous dysregulation of the c-Myc oncogene in human malignancies makes it a promising therapeutic target (145). Recent research has demonstrated that c-Myc not only regulates cell proliferation, apoptosis, and cancer metabolism, but also the TME and immune responses (145). C-Myc inhibition reprograms the cancer immune milieu by attracting T lymphocytes and activating the CD40/CD40L system in OS, according to studies (30). miR-449c has been demonstrated to directly target and negatively inhibit the production of the oncogene c-Myc, hence encouraging the advancement of the OS cell cycle (146). Her4 can boost glucose intake and tumour growth by promoting OS metabolic reprogramming *via* a c-Myc-dependent signalling pathway, suggesting that a c-Myc inhibitor may be useful in the treatment of OS (147). The S1P/S1PR3 axis has been shown to contribute to the formation of the YAP–c-Myc complex and transcription of the glycolytic enzyme PGAM1 by suppressing YAP phosphorylation and increasing its nuclear translocation, according to studies (134).

#### SGLT2 inhibitors

Sodium–glucose cotransporter 2 (SGLT2) is essential for epithelial glucose transport and is overexpressed in numerous cancer types in order to supply cancer cells with glucose to satisfy their high-energy needs (148). SGLT2 affects the expression of miR-210 and stimulates anaerobic glycolysis, hence modulating the energy metabolism of cancer cells (149). SGLT2 inhibitors significantly inhibit osteosarcoma tumour growth and induce immune cell infiltration *in vivo* by upregulating STING expression and activating the IRF3/IFN-β pathway, which could be attributable to the inhibition of AKT phosphorylation (141).

### Cancer vaccines

The protein EWS-FLI1, which is overexpressed in OS, has become a specific Treg antigen for vaccine development (150). EWS-FLI1 inhibits effector T cell responses and has been found circulating in or infiltrating tumours in Ewing patients, resulting in unfavourable clinical outcomes (150). Double sialic ganglioside (GD2) is extensively expressed in osteosarcoma (OS) and soft tissue sarcomas, and immunotherapies including GD2 vaccines have been utilized to treat solid tumor (151). miR-34a can target GD-2 to enhance tumour apoptosis, which is anticipated to be a novel OS target (152). Previous studies developed fusion cell vaccines by chemically fusing human γδT cells with SAOS-2 cells, eliciting cytotoxic T lymphocyte responses against two human OS cell lines that were specific to cancer antigens (153). CD103^+^cDC1 vaccines produced *in vitro* elicited systemic and long-lasting tumour-specific T cell-mediated cytotoxicity, thereby inhibiting the growth of primary and metastatic osteosarcoma (154).

### Engineered chimeric antigen receptor T cells

Chimeric antigen receptor T cell therapy has been shown to be effective in leukaemia and lymphoma, and current studies have increasingly focused on CAR-T therapy for solid tumours, such as OS (155). The efficacy of B7-H3-CAR-T cell therapy in treating solid tumours was initially proven in a model of childhood cancer (156). Following that, the efficacy of B7-H3-CAR-T cells in OS and preventing lung metastasis progression was demonstrated in a dose-dependent manner in a mouse model with orthotopic OS of the tibia and lung metastases (157). Human EpHA2-directed CAR-T cells can target human OS cells *in vitro*, and the injection of CAR-T cells can eradicate tumour deposits in the liver and lungs of metastatic OS models *in vivo* (158). CD166 is selectively expressed in OS cells and can be used as a new target for CAR-T cell therapy, which has been demonstrated in mice models of OS by injection of CD166.BBζ CAR-T cells (159). Human epidermal growth factor receptor 2 (HER2)-CAR-T cells have entered phase II clinical trials, and the safety and efficacy of this therapy have been demonstrated in a study of 19 patients with HER2-positive solid tumours (160).

To treat OS, immunotherapy particularly targets immune cells and immunosuppressive cells in the TME. ncRNAs play a crucial regulatory role and have the potential to be exploited as synergistic agents for checkpoint inhibitors as well as novel targets for interferon treatments and cancer vaccines. The evidence of clinical data in interferon therapy and checkpoint inhibitors is shown in **Table S2**. CAR-T cells are a new therapeutic for solid tumours that can eradicate tumour cells from primary and metastatic lesions and may provide a unique immunotherapy treatment for patients with metastatic OS.

## Discussion

The most frequent primary malignant tumour in children and adolescents is OS, which has a high rate of metastasis and a poor prognosis (161). A difference in the reduction in expression of let-7a-2 and miR-323 was identified in the differential analysis of ncRNAs in OS cells. let-7a-2 and miR-323 are regarded as sensitive prognostic indicators in a number of malignancies and may have a significant role in the clinical diagnosis of OS (162–164). The expression of circRNAs in OS is mainly decreased, of which circRNA_104892, circRNA_104893, and circRNA_104891 show significant differences in the degree of reduction. Reduced expression of circRNAs often inhibits osteosarcoma migration and invasion and promotes apoptosis, which could be combined with therapeutic targets for OS (165). lncRNA SNHG16 can function as ceRNA of miR-1285-3p to reduce the expression of miRNA, thus promote the proliferation, invasion and migration of OS cells (166). lncRNA regulates the progression of osteosarcoma through the miRNA axis, and there is no evidence for the direct regulation of lncRNA expression (167–170). lncRNA and circRNA can regulate the biological characteristics and metabolism reprogramming of OS by sponging miRNAs to represent as ceRNA (70, 94). The construction of co-expression networks of ncRNAs would be beneficial for studying OS aetiology.

OS immunotherapy primarily targets immunosuppressive cells in the TME, which are regulated by cytokines, chemokines, and an anaerobic environment (171). Gemcitabine effectively inhibited the progression of osteosarcoma by inducing cell apoptosis and inhibiting the accumulation of MDSCs (172). Additionally, when it binds to specific inhibitors of indoleamine 2, 3-dioxygenase, it can more effectively prevent the recruitment of MDSCs and the differentiation of Treg cells (172). The necessity to find novel targets has led to an increase in the number of studies on regulatory factors in OS cells. Meanwhile, when compared to a single inhibitor, a combination of inhibitors can greatly boost therapeutic efficacy. The energy uptake of immunosuppressive cells is more dependent on FAO and is also regulated by glucose levels in the TME (123, 130).

The development of combination chemotherapy has significantly increased the OS survival rate; however, the evolution of drug resistance has become a critical issue that must be addressed (173). Immunotherapy is a new strategy in the treatment of OS that targets immune cells to activate the immune system and relies on autoimmune responses to fight tumour tissues, an approach that may also be useful in combatting drug resistance (4, 173). Multiple types of checkpoint inhibitors have shown significant anticancer efficacy. The synergistic effects of checkpoint inhibitors and their combination with chemotherapy are promising options for combating drug resistance (4). Therapies based on OS-related antibodies have shown promise when combined with checkpoint inhibitors (154). In recent years, CAR-T cell treatment for OS has demonstrated encouraging results. (HER2)-CAR-T cells have entered phase II clinical trials and are expected to advance the treatment of OS (160).

In conclusion, this review summarizes the role of ncRNAs in OS cells, including their differential expression, as well as the metabolic heterogeneity of immunosuppressive cells in the TME. Emerging immunotherapies have been studied and compared in recent years, and their roles in the clinical diagnosis and treatment of OS have been investigated.

## Author contributions

YX and DoW wrote the manuscript. YP, MC, DuW, ZJ and BL collected the references and prepared figures. All authors contributed to the article and approved the submitted version.

## Funding

This research was financially supported by the National Natural Science Foundation of China (Grant Nos. 52022095, 82071391, 51973216, 51873207, and 51833010), the Provincial Health Special Project of Jilin Province (Grant Nos. JLSCZD2019-002 and JLSWSRCZX2020-095), the Science and Technology Development Program of Jilin Province (Grant No. 20200404182YY), the Youth Innovation Promotion Association of Chinese Academy of Sciences (Grant No. 2019230), and Natural science Foundation of the Jilin province (Grant No. 20210101310JC), and Jilin Provincial Science and Technology Department Project (20220505033ZP).

## Conflict of interest

The authors declare that the research was conducted in the absence of any commercial or financial relationships that could be construed as a potential conflict of interest.

## Publisher’s note

All claims expressed in this article are solely those of the authors and do not necessarily represent those of their affiliated organizations, or those of the publisher, the editors and the reviewers. Any product that may be evaluated in this article, or claim that may be made by its manufacturer, is not guaranteed or endorsed by the publisher.

## Supplementary material

Supplementary Figure 1 The ceRNA network in OS cells.

## References

[1] Teicher BA. Searching for molecular targets in sarcoma. Biochem Pharmacol (2012) 84(1):1–10. doi: 10.1016/j.bcp.2012.02.009 22387046

[2] Du X Yang J Yang D Tian W Zhu Z. The Genetic Basis for Inactivation of Wnt Pathway in Human Osteosarcoma. BMC Cancer (2014) 14:450. doi: 10.1186/1471-2407-14-450 24942472 PMC4074405

[3] Cersosimo F Lonardi S Bernardini G Telfer B Mandelli GE Santucci A. Tumor-Associated Macrophages in Osteosarcoma: From Mechanisms to Therapy. Int J Mol Sci (2020) 21(15). doi: 10.3390/ijms21155207 PMC7432207 32717819

[4] Chen C Xie L Ren T Huang Y Xu J Guo W. Immunotherapy for Osteosarcoma: Fundamental Mechanism, Rationale, and Recent Breakthroughs. Cancer Lett (2021) 500:1–10. doi: 10.1016/j.canlet.2020.12.024 33359211

[5] Bejarano L Jordao MJC Joyce JA. Therapeutic Targeting of the Tumor Microenvironment. Cancer Discov (2021) 11(4):933–59. doi: 10.1158/2159-8290.CD-20-1808 33811125

[6] Hanahan D Coussens LM. Accessories to the crime: functions of cells recruited to the tumor microenvironment. Cancer Cell. (2012) 21(3):309–22. doi: 10.1016/j.ccr.2012.02.022 22439926

[7] Pitt JM Marabelle A Eggermont A Soria JC Kroemer G Zitvogel L. Targeting the Tumor Microenvironment: Removing Obstruction to Anticancer Immune Responses and Immunotherapy. Ann Oncol (2016) 27(8):1482–92. doi: 10.1093/annonc/mdw168 27069014

[8] Mao X Xu J Wang W Liang C Hua J Liu J. Crosstalk between Cancer-Associated Fibroblasts and Immune Cells in the Tumor Microenvironment: New Findings and Future Perspectives. Mol Cancer (2021) 20(1):131. doi: 10.1186/s12943-021-01428-1 34635121 PMC8504100

[9] Lin Z Xie X Lu S Liu T. Noncoding Rnas in Osteosarcoma: Implications for Drug Resistance. Cancer Lett (2021) 504:91–103. doi: 10.1016/j.canlet.2021.02.007 33587978

[10] Wang JY Yang Y Ma Y Wang F Xue A Zhu J. Potential Regulatory Role of Lncrna-Mirna-Mrna Axis in Osteosarcoma. BioMed Pharmacother (2020) 121:109627. doi: 10.1016/j.biopha.2019.109627 31810120

[11] Zhou Y Li X Yang H. Linc00612 Functions as a Cerna for Mir-214-5p to Promote the Proliferation and Invasion of Osteosarcoma *in vitro* and *in vivo* . Exp Cell Res (2020) 392(1):112012. doi: 10.1016/j.yexcr.2020.112012 32311343

[12] Zhu T Fan D Ye K Liu B Cui Z Liu Z. Role of Mirna-542-5p in the Tumorigenesis of Osteosarcoma. FEBS Open Bio (2020) 10(4):627–36. doi: 10.1002/2211-5463.12824 PMC7137799 32105410

[13] Sun Y Cao L Lin JT Yuan Y Cao ZL Jia JD. Upregulated Mirna-1236-3p in Osteosarcoma Inhibits Cell Proliferation and Induces Apoptosis *Via* Targeting Klf8. Eur Rev Med Pharmacol Sci (2019) 23(14):6053–61. doi: 10.26355/eurrev_201907_18418 31364106

[14] Zheng S Jiang F Ge D Tang J Chen H Yang J. Lncrna Snhg3/Mirna-151a-3p/Rab22a Axis Regulates Invasion and Migration of Osteosarcoma. BioMed Pharmacother (2019) 112:108695. doi: 10.1016/j.biopha.2019.108695 30797154

[15] Wang C Ren M Zhao X Wang A Wang J. Emerging Roles of Circular Rnas in Osteosarcoma. Med Sci Monit (2018) 24:7043–50. doi: 10.12659/MSM.912092 PMC6183101 30282962

[16] Huang Y Zhou Z Zhang J Hao Z He Y Wu Z. Lncrna Malat1 Participates in Metformin Inhibiting the Proliferation of Breast Cancer Cell. J Cell Mol Med (2021) 25(15):7135–45. doi: 10.1111/jcmm.16742 PMC8335702 34164906

[17] Yahiro K Matsumoto Y. Immunotherapy for Osteosarcoma. Hum Vaccin Immunother (2021) 17(5):1294–5. doi: 10.1080/21645515.2020.1824499 PMC8078647 33356848

[18] Wang Z Li B Ren Y Ye Z. T-Cell-Based Immunotherapy for Osteosarcoma: Challenges and Opportunities. Front Immunol (2016) 7:353. doi: 10.3389/fimmu.2016.00353 27683579 PMC5021687

[19] Li Z Li X Xu D Chen X Li S Zhang L. An Update on the Roles of Circular Rnas in Osteosarcoma. Cell Prolif (2021) 54(1):e12936. doi: 10.1111/cpr.12936 33103338 PMC7791175

[20] Czarnecka AM Synoradzki K Firlej W Bartnik E Sobczuk P Fiedorowicz M. Molecular Biology of Osteosarcoma. Cancers (Basel) (2020) 12(8). doi: 10.3390/cancers12082130 PMC7463657 32751922

[21] Wang J Liu S Shi J Li J Wang S Liu H. The Role of Mirna in the Diagnosis, Prognosis, and Treatment of Osteosarcoma. Cancer Biother Radiopharm (2019) 34(10):605–13. doi: 10.1089/cbr.2019.2939 31674804

[22] Ji Q Xu X Song Q Xu Y Tai Y Goodman SB. Mir-223-3p Inhibits Human Osteosarcoma Metastasis and Progression by Directly Targeting Cdh6. Mol Ther (2018) 26(5):1299–312. doi: 10.1016/j.ymthe.2018.03.009 PMC5993963 29628305

[23] Luo P Zhang YD He F Tong CJ Liu K Liu H. Hif-1alpha-Mediated Augmentation of Mirna-18b-5p Facilitates Proliferation and Metastasis in Osteosarcoma through Attenuation Phf2. Sci Rep (2022) 12(1):10398. doi: 10.1038/s41598-022-13660-w 35729160 PMC9213540

[24] Liu X Cui M. Mirna-98-5p Inhibits the Progression of Osteosarcoma by Regulating Cell Cycle *Via* Targeting Cdc25a Expression. Eur Rev Med Pharmacol Sci (2019) 23(22):9793–802. doi: 10.26355/eurrev_201911_19542 31799646

[25] Ding L Liu T Qu Y Kang Z Guo L Zhang H. Lncrna Meltf-As1 Facilitates Osteosarcoma Metastasis by Modulating Mmp14 Expression. Mol Ther Nucleic Acids (2021) 26:787–97. doi: 10.1016/j.omtn.2021.08.022 PMC8526484 34729248

[26] Zhu KP Ma XL Zhang CL. Lncrna Odrul Contributes to Osteosarcoma Progression through the Mir-3182/Mmp2 Axis. Mol Ther (2017) 25(10):2383–93. doi: 10.1016/j.ymthe.2017.06.027 PMC5628795 28750740

[27] Zhang M Wang Y Jiang L Song X Zheng A Gao H. Lncrna Cbr3-As1 Regulates of Breast Cancer Drug Sensitivity as a Competing Endogenous Rna through the Jnk1/Mek4-Mediated Mapk Signal Pathway. J Exp Clin Cancer Res (2021) 40(1):41. doi: 10.1186/s13046-021-01844-7 33494806 PMC7830819

[28] Dai S Li N Zhou M Yuan Y Yue D Li T. Lncrna Ebln3p Promotes the Progression of Osteosarcoma through Modifying the Mir-224-5p/Rab10 Signaling Axis. Sci Rep (2021) 11(1):1992. doi: 10.1038/s41598-021-81641-6 33479458 PMC7820338

[29] Shen S Yao T Xu Y Zhang D Fan S Ma J. Circece1 Activates Energy Metabolism in Osteosarcoma by Stabilizing C-Myc. Mol Cancer (2020) 19(1):151. doi: 10.1186/s12943-020-01269-4 33106166 PMC7586679

[30] Jiang K Zhang Q Fan Y Li J Zhang J Wang W. Myc Inhibition Reprograms Tumor Immune Microenvironment by Recruiting T Lymphocytes and Activating the Cd40/Cd40l System in Osteosarcoma. Cell Death Discovery (2022) 8(1):117. doi: 10.1038/s41420-022-00923-8 35292660 PMC8924240

[31] Yang B Li L Tong G Zeng Z Tan J Su Z. Circular Rna Circ_001422 Promotes the Progression and Metastasis of Osteosarcoma *Via* the Mir-195-5p/Fgf2/Pi3k/Akt Axis. J Exp Clin Cancer Res (2021) 40(1):235. doi: 10.1186/s13046-021-02027-0 34271943 PMC8283840

[32] Chen J Liu G Wu Y Ma J Wu H Xie Z. Circmyo10 Promotes Osteosarcoma Progression by Regulating Mir-370-3p/Ruvbl1 Axis to Enhance the Transcriptional Activity of Beta-Catenin/Lef1 Complex *Via* Effects on Chromatin Remodeling. Mol Cancer (2019) 18(1):150. doi: 10.1186/s12943-019-1076-1 31665067 PMC6819556

[33] Liu Y Wang Y Yang H Zhao L Song R Tan H. Microrna873 Targets Hoxa9 to Inhibit the Aggressive Phenotype of Osteosarcoma by Deactivating the Wnt/Betacatenin Pathway. Int J Oncol (2019) 54(5):1809–20. doi: 10.3892/ijo.2019.4735 30816476

[34] Zhu R Li X Ma Y. Mir-23b-3p Suppressing Pgc1alpha Promotes Proliferation through Reprogramming Metabolism in Osteosarcoma. Cell Death Dis (2019) 10(6):381. doi: 10.1038/s41419-019-1614-1 31097683 PMC6522531

[35] Cai W Jiang H Yu Y Xu Y Zuo W Wang S. Mir-367 Regulation of Doc-2/Dab2 Interactive Protein Promotes Proliferation, Migration and Invasion of Osteosarcoma Cells. BioMed Pharmacother (2017) 95:120–8. doi: 10.1016/j.biopha.2017.07.158 28837878

[36] Sekar D Mani P Biruntha M Sivagurunathan P Karthigeyan M. Dissecting the Functional Role of Microrna 21 in Osteosarcoma. Cancer Gene Ther (2019) 26(7-8):179–82. doi: 10.1038/s41417-019-0092-z 30905966

[37] Jiang R Zhang C Liu G Gu R Wu H. Microrna-107 Promotes Proliferation, Migration, and Invasion of Osteosarcoma Cells by Targeting Tropomyosin 1. Oncol Res (2017) 25(8):1409–19. doi: 10.3727/096504017X14882829077237 PMC7841194 28276320

[38] Wang WT Qi Q Zhao P Li CY Yin XY Yan RB. Mir-590-3p Is a Novel Microrna Which Suppresses Osteosarcoma Progression by Targeting Sox9. BioMed Pharmacother (2018) 107:1763–9. doi: 10.1016/j.biopha.2018.06.124 30257395

[39] Zhang B Yu L Han N Hu Z Wang S Ding L. Linc01116 Targets Mir-520a-3p and Affects Il6r to Promote the Proliferation and Migration of Osteosarcoma Cells through the Jak-Stat Signaling Pathway. BioMed Pharmacother (2018) 107:270–82. doi: 10.1016/j.biopha.2018.07.119 30098545

[40] Wang SN Luo S Liu C Piao Z Gou W Wang Y. Mir-491 Inhibits Osteosarcoma Lung Metastasis and Chemoresistance by Targeting Alphab-Crystallin. Mol Ther (2017) 25(9):2140–9. doi: 10.1016/j.ymthe.2017.05.018 PMC5589150 28648665

[41] Shekhar R Priyanka P Kumar P Ghosh T Khan MM Nagarajan P. The Micrornas Mir-449a and Mir-424 Suppress Osteosarcoma by Targeting Cyclin A2 Expression. J Biol Chem (2019) 294(12):4381–400. doi: 10.1074/jbc.RA118.005778 PMC6433048 30679313

[42] Vimalraj S Subramanian R Saravanan S Arumugam B Anuradha D. Microrna-432-5p Regulates Sprouting and Intussusceptive Angiogenesis in Osteosarcoma Microenvironment by Targeting Pdgfb. Lab Invest (2021) 101(8):1011–25. doi: 10.1038/s41374-021-00589-3 33846539

[43] Yang G Zhang C Wang N Chen J. Mir-425-5p Decreases Lncrna Malat1 and Tug1 Expressions and Suppresses Tumorigenesis in Osteosarcoma *Via* Wnt/Beta-Catenin Signaling Pathway. Int J Biochem Cell Biol (2019) 111:42–51. doi: 10.1016/j.biocel.2019.04.004 30986552

[44] Xia P Gu R Zhang W Shao L Li F Wu C. Microrna-377 Exerts a Potent Suppressive Role in Osteosarcoma through the Involvement of the Histone Acetyltransferase 1-Mediated Wnt Axis. J Cell Physiol (2019) 234(12):22787–98. doi: 10.1002/jcp.28843 31152456

[45] Wang K Yan L Lu F. Mir-363-3p Inhibits Osteosarcoma Cell Proliferation and Invasion *Via* Targeting Sox4. Oncol Res (2019) 27(2):157–63. doi: 10.3727/096504018X15190861873459 PMC7848441 29471893

[46] Zhang S Liu L Lv Z Li Q Gong W Wu H. Microrna-342-3p Inhibits the Proliferation, Migration, and Invasion of Osteosarcoma Cells by Targeting Astrocyte-Elevated Gene-1 (Aeg-1). Oncol Res (2017) 25(9):1505–15. doi: 10.3727/096504017X14886485417426 PMC7841055 28276315

[47] Wang J Sun G. Foxo1-Malat1-Mir-26a-5p Feedback Loop Mediates Proliferation and Migration in Osteosarcoma Cells. Oncol Res (2017) 25(9):1517–27. doi: 10.3727/096504017X14859934460780 PMC7841132 28160461

[48] Pei Y Yao Q Li Y Zhang X Xie B. Microrna-211 Regulates Cell Proliferation, Apoptosis and Migration/Invasion in Human Osteosarcoma *Via* Targeting Ezrin. Cell Mol Biol Lett (2019) 24:48. doi: 10.1186/s11658-019-0173-x 31333725 PMC6617937

[49] Ying S Jianjun H Xue Y Shuwei Y Liyuan Z Jie W. Microrna-133b Inhibits Cell Proliferation and Invasion in Osteosarcoma by Targeting Sirt1. Oncol Res (2017) 25(9):1421–30. doi: 10.3727/096504016X14826089198805 PMC7841126 28059051

[50] Wang J Pang W Zuo Z Zhang W He W. Microrna-520b Suppresses Proliferation, Migration, and Invasion of Spinal Osteosarcoma Cells *Via* Downregulation of Frizzled-8. Oncol Res (2017) 25(8):1297–304. doi: 10.3727/096504017X14873430389189 PMC7841192 28247840

[51] Huang JH Xu Y Lin FY. The Inhibition of Microrna-326 by Sp1/Hdac1 Contributes to Proliferation and Metastasis of Osteosarcoma through Promoting Smo Expression. J Cell Mol Med (2020) 24(18):10876–88. doi: 10.1111/jcmm.15716 PMC7521251 32743904

[52] Duan G Zhang C Xu C Xu C Zhang L Zhang Y. Knockdown of Malat1 Inhibits Osteosarcoma Progression *Via* Regulating the Mir34a/Cyclin D1 Axis. Int J Oncol (2019) 54(1):17–28. doi: 10.3892/ijo.2018.4600 30365098 PMC6254999

[53] Yang G Song R Wang L Wu X. Knockdown of Long Non-Coding Rna Tp73-As1 Inhibits Osteosarcoma Cell Proliferation and Invasion through Sponging Mir-142. BioMed Pharmacother (2018) 103:1238–45. doi: 10.1016/j.biopha.2018.04.146 29864904

[54] Cai L Lv J Zhang Y Li J Wang Y Yang H. The Lncrna Hnf1a-As1 Is a Negative Prognostic Factor and Promotes Tumorigenesis in Osteosarcoma. J Cell Mol Med (2017) 21(11):2654–62. doi: 10.1111/jcmm.12944 PMC5661255 28866868

[55] Yang Y Fei M Zhou X Li Y Jin D. The Potential Value of Lncrna-Bc050642 in Osteosarcoma Origination and Outcomes. Artif Cells Nanomed Biotechnol (2019) 47(1):1859–66. doi: 10.1080/21691401.2019.1611593 31397185

[56] Dai J Xu LJ Han GD Jiang HT Sun HL Zhu GT. Down-Regulation of Long Non-Coding Rna Itgb2-As1 Inhibits Osteosarcoma Proliferation and Metastasis by Repressing Wnt/Beta-Catenin Signalling and Predicts Favourable Prognosis. Artif Cells Nanomed Biotechnol (2018) 46(sup3):S783–S90. doi: 10.1080/21691401.2018.1511576 30260245

[57] Lee AM Ferdjallah A Moore E Kim DC Nath A Greengard E. Long Non-Coding Rna Anril as a Potential Biomarker of Chemosensitivity and Clinical Outcomes in Osteosarcoma. Int J Mol Sci (2021) 22(20). doi: 10.3390/ijms222011168 PMC8538287 34681828

[58] Lv GY Miao J Zhang XL. Long Noncoding Rna Xist Promotes Osteosarcoma Progression by Targeting Ras-Related Protein Rap2b *Via* Mir-320b. Oncol Res (2018) 26(6):837–46. doi: 10.3727/096504017X14920318811721 PMC7844768 28409547

[59] Li Y Zhang T Zhang Y Zhao X Wang W. Targeting the Foxm1-Regulated Long Noncoding Rna Tug1 in Osteosarcoma. Cancer Sci (2018) 109(10):3093–104. doi: 10.1111/cas.13765 PMC6172046 30099814

[60] Yu X Hu L Li S Shen J Wang D Xu R. Long Non-Coding Rna Taurine Upregulated Gene 1 Promotes Osteosarcoma Cell Metastasis by Mediating Hif-1alpha *Via* Mir-143-5p. Cell Death Dis (2019) 10(4):280. doi: 10.1038/s41419-019-1509-1 30911001 PMC6433912

[61] Yao W Yan Q Du X Hou J. Tnk2-As1 Upregulated by Yy1 Boosts the Course of Osteosarcoma through Targeting Mir-4319/Wdr1. Cancer Sci (2021) 112(2):893–905. doi: 10.1111/cas.14727 33164271 PMC7893995

[62] Xu R Feng F Yu X Liu Z Lao L. Lncrna Snhg4 Promotes Tumour Growth by Sponging Mir-224-3p and Predicts Poor Survival and Recurrence in Human Osteosarcoma. Cell Prolif (2018) 51(6):e12515. doi: 10.1111/cpr.12515 30152090 PMC6528889

[63] Wang J Cao L Wu J Wang Q. Long Non-Coding Rna Snhg1 Regulates Nob1 Expression by Sponging Mir-326 and Promotes Tumorigenesis in Osteosarcoma. Int J Oncol (2018) 52(1):77–88. doi: 10.3892/ijo.2017.4187 29115574 PMC5743365

[64] Wang X Hu K Chao Y Wang L. Lncrna Snhg16 Promotes Proliferation, Migration and Invasion of Osteosarcoma Cells by Targeting Mir-1301/Bcl9 Axis. BioMed Pharmacother (2019) 114:108798. doi: 10.1016/j.biopha.2019.108798 30909141

[65] Sun X Tian C Zhang H Han K Zhou M Gan Z. Long Noncoding Rna Oip5-As1 Mediates Resistance to Doxorubicin by Regulating Mir-137-3p/Ptn Axis in Osteosarcoma. BioMed Pharmacother (2020) 128:110201. doi: 10.1016/j.biopha.2020.110201 32460190

[66] Dai J Xu L Hu X Han G Jiang H Sun H. Long Noncoding Rna Oip5-As1 Accelerates Cdk14 Expression to Promote Osteosarcoma Tumorigenesis *Via* Targeting Mir-223. BioMed Pharmacother (2018) 106:1441–7. doi: 10.1016/j.biopha.2018.07.109 30119217

[67] Su X Teng J Jin G Li J Zhao Z Cao X. Elk1-Induced Upregulation of Long Non-Coding Rna Mir100hg Predicts Poor Prognosis and Promotes the Progression of Osteosarcoma by Epigenetically Silencing Lats1 and Lats2. BioMed Pharmacother (2019) 109:788–97. doi: 10.1016/j.biopha.2018.10.029 30551532

[68] Pan X Tan J Tao T Zhang X Weng Y Weng X. Linc01123 Enhances Osteosarcoma Cell Growth by Activating the Hedgehog Pathway *Via* the Mir-516b-5p/Gli1 Axis. Cancer Sci (2021) 112(6):2260–71. doi: 10.1111/cas.14913 PMC8177773 33837611

[69] Wu S Gu Z Wu Y Wu W Mao B Zhao S. Linc00324 Accelerates the Proliferation and Migration of Osteosarcoma through Regulating Wdr66. J Cell Physiol (2020) 235(1):339–48. doi: 10.1002/jcp.28973 31225659

[70] Shen Y Xu J Pan X Zhang Y Weng Y Zhou D. Lncrna Kcnq1ot1 Sponges Mir-34c-5p to Promote Osteosarcoma Growth *Via* Aldoa Enhanced Aerobic Glycolysis. Cell Death Dis (2020) 11(4):278. doi: 10.1038/s41419-020-2485-1 32332718 PMC7181648

[71] Li Y Zou J Li B Du J. Anticancer Effects of Melatonin *Via* Regulating Lncrna Jpx-Wnt/Beta-Catenin Signalling Pathway in Human Osteosarcoma Cells. J Cell Mol Med (2021) 25(20):9543–56. doi: 10.1111/jcmm.16894 PMC8505851 34547170

[72] Li Y Liu JJ Zhou JH Chen R Cen CQ. Lncrna Hulc Induces the Progression of Osteosarcoma by Regulating the Mir-372-3p/Hmgb1 Signalling Axis. Mol Med (2020) 26(1):26. doi: 10.1186/s10020-020-00155-5 32188407 PMC7081592

[73] Qu Y Zheng S Kang M Dong R Zhou H Zhao D. Knockdown of Long Non-Coding Rna Hoxd-As1 Inhibits the Progression of Osteosarcoma. BioMed Pharmacother (2018) 98:899–906. doi: 10.1016/j.biopha.2018.01.024 29571260

[74] Gu W Zhang E Song L Tu L Wang Z Tian F. Long Noncoding Rna Hoxd-As1 Aggravates Osteosarcoma Carcinogenesis through Epigenetically Inhibiting P57 *Via* Ezh2. BioMed Pharmacother (2018) 106:890–5. doi: 10.1016/j.biopha.2018.06.173 30119259

[75] Ren Z Hu Y Li G Kang Y Liu Y Zhao H. Hif-1alpha Induced Long Noncoding Rna Foxd2-As1 Promotes the Osteosarcoma through Repressing P21. BioMed Pharmacother (2019) 117:109104. doi: 10.1016/j.biopha.2019.109104 31228799

[76] Chen X Zhang C Wang X. Long Noncoding Rna Dleu1 Aggravates Osteosarcoma Carcinogenesis *Via* Regulating the Mir-671-5p/Ddx5 Axis. Artif Cells Nanomed Biotechnol (2019) 47(1):3322–8. doi: 10.1080/21691401.2019.1648285 31379208

[77] Wang Y Zeng X Wang N Zhao W Zhang X Teng S. Long Noncoding Rna Dancr, Working as a Competitive Endogenous Rna, Promotes Rock1-Mediated Proliferation and Metastasis *Via* Decoying of Mir-335-5p and Mir-1972 in Osteosarcoma. Mol Cancer (2018) 17(1):89. doi: 10.1186/s12943-018-0837-6 29753317 PMC5948795

[78] Jiang N Wang X Xie X Liao Y Liu N Liu J. Lncrna Dancr Promotes Tumor Progression and Cancer Stemness Features in Osteosarcoma by Upregulating Axl *Via* Mir-33a-5p Inhibition. Cancer Lett (2017) 405:46–55. doi: 10.1016/j.canlet.2017.06.009 28642170

[79] Yan L Wu X Yin X Du F Liu Y Ding X. Lncrna Ccat2 Promoted Osteosarcoma Cell Proliferation and Invasion. J Cell Mol Med (2018) 22(5):2592–9. doi: 10.1111/jcmm.13518 PMC5908115 29502343

[80] Zhang Y Meng W Cui H. Lncrna Cbr3-As1 Predicts Unfavorable Prognosis and Promotes Tumorigenesis in Osteosarcoma. BioMed Pharmacother (2018) 102:169–74. doi: 10.1016/j.biopha.2018.02.081 29554595

[81] Guan H Shang G Cui Y Liu J Sun X Cao W. Long Noncoding Rna Aptr Contributes to Osteosarcoma Progression through Repression of Mir-132-3p and Upregulation of Yes-Associated Protein 1. J Cell Physiol (2019) 234(6):8998–9007. doi: 10.1002/jcp.27572 30317613

[82] Xia B Wang L Feng L Tian B Tan Y Du B. Knockdown of Long Noncoding Rna Cat104 Inhibits the Proliferation, Migration, and Invasion of Human Osteosarcoma Cells by Regulating Microrna-381. Oncol Res (2018) 27(1):89–98. doi: 10.3727/096504018X15199511344806 29523223 PMC7848448

[83] Yao Q Chen T. Linc01128 Regulates the Development of Osteosarcoma by Sponging Mir-299-3p to Mediate Mmp2 Expression and Activating Wnt/Beta-Catenin Signalling Pathway. J Cell Mol Med (2020) 24(24):14293–305. doi: 10.1111/jcmm.16046 PMC7753992 33108067

[84] Zhang L Wang Y Zhang L Xia X Chao Y He R. Zbtb7a, a Mir-663a Target Gene, Protects Osteosarcoma from Endoplasmic Reticulum Stress-Induced Apoptosis by Suppressing Lncrna Gas5 Expression. Cancer Lett (2019) 448:105–16. doi: 10.1016/j.canlet.2019.01.046 30753838

[85] Wu D Nie X Ma C Liu X Liang X An Y. Rsf1 Functions as an Oncogene in Osteosarcoma and Is Regulated by Xist/Mir-193a-3p Axis. BioMed Pharmacother (2017) 95:207–14. doi: 10.1016/j.biopha.2017.08.068 28843909

[86] Hu R Zhu X Chen C Xu R Li Y Xu W. Rna-Binding Protein Pum2 Suppresses Osteosarcoma Progression *Via* Partly and Competitively Binding to Stard13 3'utr with Mirnas. Cell Prolif (2018) 51(6):e12508. doi: 10.1111/cpr.12508 30084199 PMC6528862

[87] Zhang R Xia T. Long Non-Coding Rna Xist Regulates Pdcd4 Expression by Interacting with Mir-21-5p and Inhibits Osteosarcoma Cell Growth and Metastasis. Int J Oncol (2017) 51(5):1460–70. doi: 10.3892/ijo.2017.4127 PMC5643066 29048648

[88] Jia D Niu Y Li D Liu Z. Lncrna C2dat1 Promotes Cell Proliferation, Migration, and Invasion by Targeting Mir-34a-5p in Osteosarcoma Cells. Oncol Res (2018) 26(5):753–64. doi: 10.3727/096504017X15024946480113 PMC7844639 28810936

[89] Li S Zeng M Yang L Tan J Yang J Guan H. Hsa_Circ_0008934 Promotes the Proliferation and Migration of Osteosarcoma Cells by Targeting Mir-145-5p to Enhance E2f3 Expression. Int J Biochem Cell Biol (2020) 127:105826. doi: 10.1016/j.biocel.2020.105826 32822848

[90] Li B Li X. Overexpression of Hsa_Circ_0007534 Predicts Unfavorable Prognosis for Osteosarcoma and Regulates Cell Growth and Apoptosis by Affecting Akt/Gsk-3beta Signaling Pathway. BioMed Pharmacother (2018) 107:860–6. doi: 10.1016/j.biopha.2018.08.086 30142548

[91] Lou J Zhang H Xu J Ren T Huang Y Tang X. Circusp34 Accelerates Osteosarcoma Malignant Progression by Sponging Mir-16-5p. Cancer Sci (2022) 113(1):120–31. doi: 10.1111/cas.15147 PMC8748222 34592064

[92] Yu Y Dong G Li Z Zheng Y Shi Z Wang G. Circlrp6 Contributes to Osteosarcoma Progression by Regulating the Mir1413p/Hdac4/Hmgb1 Axis. Int J Oncol (2022) 60. doi: 10.3892/ijo.2022.5328 PMC8878724 35211755

[93] Ma W Xue N Zhang J Wang D Yao X Lin L. Circubap2 Regulates Osteosarcoma Progression *Via* the Mir2043p/Hmga2 Axis. Int J Oncol (2021) 58(3):298–311. doi: 10.3892/ijo.2021.5178 33650644 PMC7864148

[94] Wu Y Xie Z Chen J Chen J Ni W Ma Y. Circular Rna Circtada2a Promotes Osteosarcoma Progression and Metastasis by Sponging Mir-203a-3p and Regulating Creb3 Expression. Mol Cancer (2019) 18(1):73. doi: 10.1186/s12943-019-1007-1 30940151 PMC6444890

[95] Zhang M Yu GY Liu G Liu WD. Circular Rna Circ_0002137 Regulated the Progression of Osteosarcoma through Regulating Mir-433-3p/ Igf1r Axis. J Cell Mol Med (2022) 26(6):1806–16. doi: 10.1111/jcmm.16166 PMC8918411 33621401

[96] Wan J Liu Y Long F Tian J Zhang C. Circpvt1 Promotes Osteosarcoma Glycolysis and Metastasis by Sponging Mir-423-5p to Activate Wnt5a/Ror2 Signaling. Cancer Sci (2021) 112(5):1707–22. doi: 10.1111/cas.14787 PMC8088910 33369809

[97] Zhang G Zhu Y Jin C Shi Q An X Song L. Circrna_0078767 Promotes Osteosarcoma Progression by Increasing Cdk14 Expression through Sponging Microrna-330-3p. Chem Biol Interact (2022) 360:109903. doi: 10.1016/j.cbi.2022.109903 35307379

[98] Ji X Shan L Shen P He M. Circular Rna Circ_001621 Promotes Osteosarcoma Cells Proliferation and Migration by Sponging Mir-578 and Regulating Vegf Expression. Cell Death Dis (2020) 11(1):18. doi: 10.1038/s41419-019-2204-y 31907361 PMC6944700

[99] Li L Guo L Yin G Yu G Zhao Y Pan Y. Upregulation of Circular Rna Circ_0001721 Predicts Unfavorable Prognosis in Osteosarcoma and Facilitates Cell Progression *Via* Sponging Mir-569 and Mir-599. BioMed Pharmacother (2019) 109:226–32. doi: 10.1016/j.biopha.2018.10.072 30396080

[100] Jiang X Chen D. Circular Rna Hsa_Circ_0000658 Inhibits Osteosarcoma Cell Proliferation and Migration *Via* the Mir-1227/Irf2 Axis. J Cell Mol Med (2021) 25(1):510–20. doi: 10.1111/jcmm.16105 PMC7810968 33264494

[101] Li S Pei Y Wang W Liu F Zheng K Zhang X. Extracellular Nanovesicles-Transmitted Circular Rna Has_Circ_0000190 Suppresses Osteosarcoma Progression. J Cell Mol Med (2020) 24(3):2202–14. doi: 10.1111/jcmm.14877 PMC7011131 31923350

[102] Palmini G Marini F Brandi ML. What Is New in the Mirna World Regarding Osteosarcoma and Chondrosarcoma? Molecules (2017) 22(3). doi: 10.3390/molecules22030417 PMC6155266 28272374

[103] Zhu KP Zhang CL Ma XL Hu JP Cai T Zhang L. Analyzing the Interactions of Mrnas and Ncrnas to Predict Competing Endogenous Rna Networks in Osteosarcoma Chemo-Resistance. Mol Ther (2019) 27(3):518–30. doi: 10.1016/j.ymthe.2019.01.001 PMC6401193 30692017

[104] Draghiciu O Lubbers J Nijman HW Daemen T. Myeloid Derived Suppressor Cells-an Overview of Combat Strategies to Increase Immunotherapy Efficacy. Oncoimmunology (2015) 4(1):e954829. doi: 10.4161/21624011.2014.954829 25949858 PMC4368153

[105] Coussens LM Werb Z. Inflammation and Cancer. Nature (2002) 420(6917):860–7. doi: 10.1038/nature01322 PMC2803035 12490959

[106] Ling Z Yang C Tan J Dou C Chen Y. Beyond Immunosuppressive Effects: Dual Roles of Myeloid-Derived Suppressor Cells in Bone-Related Diseases. Cell Mol Life Sci (2021) 78(23):7161–83. doi: 10.1007/s00018-021-03966-9 PMC11072300 34635950

[107] Zhao Q Huang L Qin G Qiao Y Ren F Shen C. Cancer-Associated Fibroblasts Induce Monocytic Myeloid-Derived Suppressor Cell Generation *Via* Il-6/Exosomal Mir-21-Activated Stat3 Signaling to Promote Cisplatin Resistance in Esophageal Squamous Cell Carcinoma. Cancer Lett (2021) 518:35–48. doi: 10.1016/j.canlet.2021.06.009 34139285

[108] Kirtonia A Sethi G Garg M. The Multifaceted Role of Reactive Oxygen Species in Tumorigenesis. Cell Mol Life Sci (2020) 77(22):4459–83. doi: 10.1007/s00018-020-03536-5 PMC11105050 32358622

[109] Ohl K Tenbrock K. Reactive Oxygen Species as Regulators of Mdsc-Mediated Immune Suppression. Front Immunol (2018) 9:2499. doi: 10.3389/fimmu.2018.02499 30425715 PMC6218613

[110] Veglia F Sanseviero E Gabrilovich DI. Myeloid-Derived Suppressor Cells in the Era of Increasing Myeloid Cell Diversity. Nat Rev Immunol (2021) 21(8):485–98. doi: 10.1038/s41577-020-00490-y PMC7849958 33526920

[111] Wang B Tontonoz P. Liver X Receptors in Lipid Signalling and Membrane Homeostasis. Nat Rev Endocrinol (2018) 14(8):452–63. doi: 10.1038/s41574-018-0037-x PMC6433546 29904174

[112] Tavazoie MF Pollack I Tanqueco R Ostendorf BN Reis BS Gonsalves FC. Lxr/Apoe Activation Restricts Innate Immune Suppression in Cancer. Cell (2018) 172(4):825–40.e18. doi: 10.1016/j.cell.2017.12.026 29336888 PMC5846344

[113] Rodriguez PC Ochoa AC Al-Khami AA. Arginine Metabolism in Myeloid Cells Shapes Innate and Adaptive Immunity. Front Immunol (2017) 8:93. doi: 10.3389/fimmu.2017.00093 28223985 PMC5293781

[114] Satoh Y Kotani H Iida Y Taniura T Notsu Y Harada M. Supplementation of L-Arginine Boosts the Therapeutic Efficacy of Anticancer Chemoimmunotherapy. Cancer Sci (2020) 111(7):2248–58. doi: 10.1111/cas.14490 PMC7484823 32426941

[115] Huang Q Liang X Ren T Huang Y Zhang H Yu Y. The Role of Tumor-Associated Macrophages in Osteosarcoma Progression - Therapeutic Implications. Cell Oncol (Dordr) (2021) 44(3):525–39. doi: 10.1007/s13402-021-00598-w PMC12980758 33788151

[116] Fujiwara T Fukushi J Yamamoto S Matsumoto Y Setsu N Oda Y. Macrophage Infiltration Predicts a Poor Prognosis for Human Ewing Sarcoma. Am J Pathol (2011) 179(3):1157–70. doi: 10.1016/j.ajpath.2011.05.034 PMC3157220 21771572

[117] Han Y Guo W Ren T Huang Y Wang S Liu K. Tumor-Associated Macrophages Promote Lung Metastasis and Induce Epithelial-Mesenchymal Transition in Osteosarcoma by Activating the Cox-2/Stat3 Axis. Cancer Lett (2019) 440-441:116–25. doi: 10.1016/j.canlet.2018.10.011 30343113

[118] Su Y Zhou Y Sun YJ Wang YL Yin JY Huang YJ. Macrophage-Derived Ccl18 Promotes Osteosarcoma Proliferation and Migration by Upregulating the Expression of Uca1. J Mol Med (Berl) (2019) 97(1):49–61. doi: 10.1007/s00109-018-1711-0 30426155

[119] He F Ding G Jiang W Fan X Zhu L. Effect of Tumor-Associated Macrophages on Lncrna Purpl/Mir-363/Pdzd2 Axis in Osteosarcoma Cells. Cell Death Discovery (2021) 7(1):307. doi: 10.1038/s41420-021-00700-z 34686652 PMC8536668

[120] Wu K Lin K Li X Yuan X Xu P Ni P. Redefining Tumor-Associated Macrophage Subpopulations and Functions in the Tumor Microenvironment. Front Immunol (2020) 11:1731. doi: 10.3389/fimmu.2020.01731 32849616 PMC7417513

[121] Liu W Long Q Zhang W Zeng D Hu B Liu S. Mirna-221-3p Derived from M2-Polarized Tumor-Associated Macrophage Exosomes Aggravates the Growth and Metastasis of Osteosarcoma through Socs3/Jak2/Stat3 Axis. Aging (Albany NY) (2021) 13(15):19760–75. doi: 10.18632/aging.203388 PMC8386545 34388111

[122] Yang D Liu K Fan L Liang W Xu T Jiang W. Lncrna Rp11-361f15.2 Promotes Osteosarcoma Tumorigenesis by Inhibiting M2-Like Polarization of Tumor-Associated Macrophages of Cpeb4. Cancer Lett (2020) 473:33–49. doi: 10.1016/j.canlet.2019.12.041 31904478

[123] Su P Wang Q Bi E Ma X Liu L Yang M. Enhanced Lipid Accumulation and Metabolism Are Required for the Differentiation and Activation of Tumor-Associated Macrophages. Cancer Res (2020) 80(7):1438–50. doi: 10.1158/0008-5472.CAN-19-2994 PMC7127942 32015091

[124] Liu S Zhang H Li Y Zhang Y Bian Y Zeng Y. S100a4 Enhances Protumor Macrophage Polarization by Control of Ppar-Gamma-Dependent Induction of Fatty Acid Oxidation. J Immunother Cancer (2021) 9(6). doi: 10.1136/jitc-2021-002548 PMC8215236 34145030

[125] Tanaka A Sakaguchi S. Regulatory T Cells in Cancer Immunotherapy. Cell Res (2017) 27(1):109–18. doi: 10.1038/cr.2016.151 PMC5223231 27995907

[126] Wing JB Tanaka A Sakaguchi S. Human Foxp3(+) Regulatory T Cell Heterogeneity and Function in Autoimmunity and Cancer. Immunity (2019) 50(2):302–16. doi: 10.1016/j.immuni.2019.01.020 30784578

[127] Biller BJ Guth A Burton JH Dow SW. Decreased Ratio of Cd8+ T Cells to Regulatory T Cells Associated with Decreased Survival in Dogs with Osteosarcoma. J Vet Intern Med (2010) 24(5):1118–23. doi: 10.1111/j.1939-1676.2010.0557.x PMC3557512 20666983

[128] Fritzsching B Fellenberg J Moskovszky L Sapi Z Krenacs T Machado I. Cd8(+)/Foxp3(+)-Ratio in Osteosarcoma Microenvironment Separates Survivors from Non-Survivors: A Multicenter Validated Retrospective Study. Oncoimmunology (2015) 4(3):e990800. doi: 10.4161/2162402X.2014.990800 25949908 PMC4404826

[129] Cluxton D Petrasca A Moran B Fletcher JM. Differential Regulation of Human Treg and Th17 Cells by Fatty Acid Synthesis and Glycolysis. Front Immunol (2019) 10:115. doi: 10.3389/fimmu.2019.00115 30778354 PMC6369198

[130] Watson MJ Vignali PDA Mullett SJ Overacre-Delgoffe AE Peralta RM Grebinoski S. Metabolic support of tumour-infiltrating regulatory T cells by lactic acid. Nature. (2021) 591(7851):645–51. doi: 10.1038/s41586-020-03045-2 PMC7990682 33589820

[131] Huynh A DuPage M Priyadharshini B Sage PT Quiros J Borges CM. Control of PI(3) kinase in Treg cells maintains homeostasis and lineage stability. Nat Immunol (2015) 16(2):188–96. doi: 10.1038/ni.3077 PMC4297515 25559257

[132] Xie M Wang J Gong W Xu H Pan X Chen Y. Nf-Kappab-Driven Mir-34a Impairs Treg/Th17 Balance *Via* Targeting Foxp3. J Autoimmun (2019) 102:96–113. doi: 10.1016/j.jaut.2019.04.018 31130368

[133] Saravia J Zeng H Dhungana Y Bastardo Blanco D Nguyen TM Chapman NM. Homeostasis and Transitional Activation of Regulatory T Cells Require C-Myc. Sci Adv (2020) 6(1):eaaw6443. doi: 10.1126/sciadv.aaw6443 31911938 PMC6938709

[134] Shen Y Zhao S Wang S Pan X Zhang Y Xu J. S1p/S1pr3 Axis Promotes Aerobic Glycolysis by Yap/C-Myc/Pgam1 Axis in Osteosarcoma. EBioMedicine (2019) 40:210–23. doi: 10.1016/j.ebiom.2018.12.038 PMC6412077 30587459

[135] Whelan J Patterson D Perisoglou M Bielack S Marina N Smeland S. The role of interferons in the treatment of osteosarcoma. Pediatr Blood Cancer. (2010) 54(3):350–4. doi: 10.1002/pbc.22136 19902521

[136] Johnson LR Lee DY Eacret JS Ye D June CH Minn AJ. The Immunostimulatory Rna Rn7sl1 Enables Car-T Cells to Enhance Autonomous and Endogenous Immune Function. Cell (2021) 184(19):4981–95 e14. doi: 10.1016/j.cell.2021.08.004 34464586 PMC11338632

[137] Xu J Ji Y Shogren KL Okuno SH Yaszemski MJ Maran A. Rna-Dependent Protein Kinase Is Required for Interferon-Gamma-Induced Autophagy in Mg63 Osteosarcoma Cells. Gene (2021) 802:145865. doi: 10.1016/j.gene.2021.145865 34352301

[138] Zhou C Zhang Y Yan R Huang L Mellor AL Yang Y. Exosome-Derived Mir-142-5p Remodels Lymphatic Vessels and Induces Ido to Promote Immune Privilege in the Tumour Microenvironment. Cell Death Differ (2021) 28(2):715–29. doi: 10.1038/s41418-020-00618-6 PMC7862304 32929219

[139] Zhu L Qiu C Dai L Zhang L Feng M Yang Y. Hsa-Mir-31 Governs T-Cell Homeostasis in Hiv Protection *Via* Ifn-Gamma-Stat1-T-Bet Axis. Front Immunol (2021) 12:771279. doi: 10.3389/fimmu.2021.771279 34804062 PMC8602903

[140] Zhang L Xue L Wu Y Wu Q Ren H Song X. Exosomes Loaded with Programmed Death Ligand-1 Promote Tumor Growth by Immunosuppression in Osteosarcoma. Bioengineered (2021) 12(2):9520–30. doi: 10.1080/21655979.2021.1996509 PMC8810114 34699324

[141] Ai L Xu A Xu J. Roles of PD-1/PD-L1 Pathway: Signaling, Cancer, and Beyond. Adv Exp Med Biol. (2020) 1248:33–59. doi: 10.1007/978-981-15-3266-5_3. 32185706

[142] Wu W Zhang Z Jing D Huang X Ren D Shao Z. Sglt2 Inhibitor Activates the Sting/Irf3/Ifn-Beta Pathway and Induces Immune Infiltration in Osteosarcoma. Cell Death Dis (2022) 13(6):523. doi: 10.1038/s41419-022-04980-w 35662245 PMC9166744

[143] Ji X Wang E Tian F. Microrna-140 Suppresses Osteosarcoma Tumor Growth by Enhancing Anti-Tumor Immune Response and Blocking Mtor Signaling. Biochem Biophys Res Commun (2018) 495(1):1342–8. doi: 10.1016/j.bbrc.2017.11.120 29170130

[144] Christie JD Appel N Canter H Achi JG Elliott NM de Matos AL. Systemic Delivery of Tnf-Armed Myxoma Virus Plus Immune Checkpoint Inhibitor Eliminates Lung Metastatic Mouse Osteosarcoma. Mol Ther Oncolytics (2021) 22:539–54. doi: 10.1016/j.omto.2021.07.014 PMC8433070 34553039

[145] Dang CV Le A Gao P. Myc-Induced Cancer Cell Energy Metabolism and Therapeutic Opportunities. Clin Cancer Res (2009) 15(21):6479–83. doi: 10.1158/1078-0432.CCR-09-0889 PMC2783410 19861459

[146] Li Q Li H Zhao X Wang B Zhang L Zhang C. DNA Methylation Mediated Downregulation of Mir-449c Controls Osteosarcoma Cell Cycle Progression by Directly Targeting Oncogene C-Myc. Int J Biol Sci (2017) 13(8):1038–50. doi: 10.7150/ijbs.19476 PMC5599909 28924385

[147] Han J Zhang Y Xu J Zhang T Wang H Wang Z. Her4 Promotes Cancer Metabolic Reprogramming *Via* the C-Myc-Dependent Signaling Axis. Cancer Lett (2021) 496:57–71. doi: 10.1016/j.canlet.2020.10.008 33038488

[148] Ren D Sun Y Zhang D Li D Liu Z Jin X. Sglt2 Promotes Pancreatic Cancer Progression by Activating the Hippo Signaling Pathway *Via* the Hnrnpk-Yap1 Axis. Cancer Lett (2021) 519:277–88. doi: 10.1016/j.canlet.2021.07.035 34314754

[149] Nakada C Hijiya N Tsukamoto Y Yano S Kai T Uchida T. A Transgenic Mouse Expressing Mir-210 in Proximal Tubule Cells Shows Mitochondrial Alteration: Possible Association of Mir-210 with a Shift in Energy Metabolism. J Pathol (2020) 251(1):12–25. doi: 10.1002/path.5394 32073141

[150] Gorthi A Romero JC Loranc E Cao L Lawrence LA Goodale E. Ews-Fli1 Increases Transcription to Cause R-Loops and Block Brca1 Repair in Ewing Sarcoma. Nature (2018) 555(7696):387–91. doi: 10.1038/nature25748 PMC6318124 29513652

[151] Nazha B Inal C Owonikoko TK. Disialoganglioside Gd2 Expression in Solid Tumors and Role as a Target for Cancer Therapy. Front Oncol (2020) 10:1000. doi: 10.3389/fonc.2020.01000 32733795 PMC7358363

[152] Tivnan A Orr WS Gubala V Nooney R Williams DE McDonagh C. Inhibition of Neuroblastoma Tumor Growth by Targeted Delivery of Microrna-34a Using Anti-Disialoganglioside Gd2 Coated Nanoparticles. PloS One (2012) 7(5):e38129. doi: 10.1371/journal.pone.0038129 22662276 PMC3360657

[153] Wang Y Zhu J Yu W Wang J Xia K Liang C. Allogenic Gammadelta T Cell and Tumor Cell Fused Vaccine for Enhanced Immunotherapeutic Efficacy of Osteosarcoma. J Bone Oncol (2020) 21:100214. doi: 10.1016/j.jbo.2018.100214 32368439 PMC7184232

[154] Zhou Y Slone N Chrisikos TT Kyrysyuk O Babcock RL Medik YB. Vaccine Efficacy against Primary and Metastatic Cancer with in Vitro-Generated Cd103(+) Conventional Dendritic Cells. J Immunother Cancer (2020) 8(1). doi: 10.1136/jitc-2019-000474 PMC7254126 32273347

[155] Lin Z Wu Z Luo W. Chimeric Antigen Receptor T-Cell Therapy: The Light of Day for Osteosarcoma. Cancers (Basel) (2021), 13(17). doi: 10.3390/cancers13174469 34503279 PMC8431424

[156] Majzner RG Theruvath JL Nellan A Heitzeneder S Cui Y Mount CW. Car T Cells Targeting B7-H3, a Pan-Cancer Antigen, Demonstrate Potent Preclinical Activity against Pediatric Solid Tumors and Brain Tumors. Clin Cancer Res (2019) 25(8):2560–74. doi: 10.1158/1078-0432.CCR-18-0432 PMC8456711 30655315

[157] Talbot LJ Chabot A Funk A Nguyen P Wagner J Ross A. A Novel Orthotopic Implantation Technique for Osteosarcoma Produces Spontaneous Metastases and Illustrates Dose-Dependent Efficacy of B7-H3-Car T Cells. Front Immunol (2021) 12:691741. doi: 10.3389/fimmu.2021.691741 34211478 PMC8239305

[158] Hsu K Middlemiss S Saletta F Gottschalk S McCowage GB Kramer B. Chimeric Antigen Receptor-Modified T Cells Targeting Epha2 for the Immunotherapy of Paediatric Bone Tumours. Cancer Gene Ther (2021) 28(3-4):321–34. doi: 10.1038/s41417-020-00221-4 PMC8057949 32873870

[159] Wang Y Yu W Zhu J Wang J Xia K Liang C. Anti-Cd166/4-1bb Chimeric Antigen Receptor T Cell Therapy for the Treatment of Osteosarcoma. J Exp Clin Cancer Res (2019) 38(1):168. doi: 10.1186/s13046-019-1147-6 30995926 PMC6471997

[160] Ahmed N Brawley VS Hegde M Robertson C Ghazi A Gerken C. Human Epidermal Growth Factor Receptor 2 (Her2) -Specific Chimeric Antigen Receptor-Modified T Cells for the Immunotherapy of Her2-Positive Sarcoma. J Clin Oncol (2015) 33(15):1688–96. doi: 10.1200/JCO.2014.58.0225 PMC4429176 25800760

[161] Sangle NA Layfield LJ. Telangiectatic Osteosarcoma. Arch Pathol Lab Med (2012) 136(5):572–6. doi: 10.5858/arpa.2011-0204-RS 22540307

[162] Wang BG Jiang LY Xu Q. A Comprehensive Evaluation for Polymorphisms in Let-7 Family in Cancer Risk and Prognosis: A System Review and Meta-Analysis. Biosci Rep (2018) 38(4). doi: 10.1042/BSR20180273 PMC6066660 29717029

[163] Liu Y Li L Liu Z Yuan Q Lu X. Plasma Mir-323 as a Biomarker for Screening Papillary Thyroid Cancer from Healthy Controls. Front Med (Lausanne) (2020) 7:122. doi: 10.3389/fmed.2020.00122 32478079 PMC7242560

[164] Jinlong S Lin F Yonghui L Li Y Weidong W. Identification of Let-7a-2-3p or/and Mir-188-5p as Prognostic Biomarkers in Cytogenetically Normal Acute Myeloid Leukemia. PloS One (2015) 10(2):e0118099. doi: 10.1371/journal.pone.0118099 25646775 PMC4315415

[165] Liu J Yang L Fu Q Liu S. Emerging Roles and Potential Biological Value of Circrna in Osteosarcoma. Front Oncol (2020) 10:552236. doi: 10.3389/fonc.2020.552236 33251132 PMC7673402

[166] Xiao X Jiang G Zhang S Hu S Fan Y Li G. Lncrna Snhg16 Contributes to Osteosarcoma Progression by Acting as a Cerna of Mir-1285-3p. BMC Cancer (2021) 21(1):355. doi: 10.1186/s12885-021-07933-2 33823834 PMC8022398

[167] Fu D Lu C Qu X Li P Chen K Shan L. Lncrna Ttn-As1 Regulates Osteosarcoma Cell Apoptosis and Drug Resistance *Via* the Mir-134-5p/Mbtd1 Axis. Aging (2019) 11(19):8374–85. doi: 10.18632/aging.102325 PMC6814585 31600142

[168] Han G Guo Q Ma N Bi W Xu M Jia J. Lncrna Bcrt1 Facilitates Osteosarcoma Progression *Via* Regulating Mir-1303/Fgf7 Axis. Aging (Albany NY) (2021) 13(11):15501–10. doi: 10.18632/aging.203106 PMC8221344 34102610

[169] Zhao A Liu W Cui X Wang N Wang Y Sun L. Lncrna Tusc7 Inhibits Osteosarcoma Progression through the Mir181a/Rassf6 Axis. Int J Mol Med (2021) 47(2):583–94. doi: 10.3892/ijmm.2020.4825 PMC7797460 33416181

[170] Wang W Li Y Zhi S Li J Miao J Ding Z. Lncrna-Ror/Microrna-185-3p/Yap1 Axis Exerts Function in Biological Characteristics of Osteosarcoma Cells. Genomics (2021) 113(1 Pt 2):450–61. doi: 10.1016/j.ygeno.2020.09.009 32898639

[171] Morales E Olson M Iglesias F Dahiya S Luetkens T Atanackovic D. Role of Immunotherapy in Ewing Sarcoma. J Immunother Cancer (2020) 8(2). doi: 10.1136/jitc-2020-000653 PMC7725096 33293354

[172] Fan Q Zuo J Tian H Huang C Nice EC Shi Z. Nanoengineering a Metal-Organic Framework for Osteosarcoma Chemo-Immunotherapy by Modulating Indoleamine-2,3-Dioxygenase and Myeloid-Derived Suppressor Cells. J Exp Clin Cancer Res (2022) 41(1):162. doi: 10.1186/s13046-022-02372-8 35501823 PMC9063269

[173] Lilienthal I Herold N. Targeting Molecular Mechanisms Underlying Treatment Efficacy and Resistance in Osteosarcoma: A Review of Current and Future Strategies. Int J Mol Sci (2020), 21(18). doi: 10.3390/ijms21186885 32961800 PMC7555161

