# Supplementary material for: Modulation of immunosuppressive cells and noncoding RNAs as immunotherapy in osteosarcoma
Source: Front Immunol. 2022 Nov 15;13:1025532. doi: 10.3389/fimmu.2022.1025532 (PMC9705758; doi:10.3389/fimmu.2022.1025532)
Supplement: Supplementary file 1 [file Table_1.docx]

**Table.S1. The detailed information of differentially expressed ncRNAs in Fig.1A-C.**

| **NcRNAs** | **Regulation** | **adj.P.Val** | **P.Value** | **t** | **B** | **logFC** | **GEO accession** |
| --- | --- | --- | --- | --- | --- | --- | --- |
| has-let-7a-2 | downregulating | 0.248 | 0.0000789 | -9.442179 | 0.5921 | -4.00036 | GSE70367 |
| has-mir-323 | downregulating | 0.974 | 0.2220755 | -1.361672 | -4.8723 | -0.446332 | GSE70368 |
| has-mir-182 | upregulating | 0.974 | 0.2136996 | 1.3902408 | -4.8423 | 0.2538836 | GSE70369 |
| ENSG00000233086.6 | upregulating | 3.11E-53 | 3.70E-55 | 18.9934 | 172.3 | 4.74961 | GSE156344 |
| ENSG00000269821 | upregulating | 9.12E-44 | 2.17E-45 | 16.5438 | 128.95 | 4.13705 | GSE156345 |
| ENSG00000234445 | upregulating | 4.40E-42 | 1.57E-43 | 16.0752 | 121.33 | 4.01988 | GSE156346 |
| ENSG00000196810 | upregulating | 3.11E-41 | 1.48E-42 | 15.8289 | 117.42 | 3.9583 | GSE156347 |
| ENSG00000245614 | downregulating | 3.51E-24 | 2.09E-25 | -11.3261 | 56.52 | -2.83229 | GSE156348 |
| hsa_circRNA_104892 | downregulating | 0.0411 | 8.81E-06 | -8.886041 | 1.24489 | -3.356682 | GSE96964 |
| hsa_circRNA_104893 | downregulating | 0.0631 | 2.709E-05 | -7.739217 | 0.81874 | -2.252041 | GSE96965 |
| has_circRNA_104891 | downregulating | 0.1305 | 0.000084 | -6.701009 | 0.32018 | -2.022954 | GSE96966 |
