# Supplementary material for: Modulation of immunosuppressive cells and noncoding RNAs as immunotherapy in osteosarcoma
Source: Front Immunol. 2022 Nov 15;13:1025532. doi: 10.3389/fimmu.2022.1025532 (PMC9705758; doi:10.3389/fimmu.2022.1025532)
Supplement: Supplementary file 2 [file Table_2.docx]

**Table S2. Clinical application of interferon therapies and checkpoint inhibitors in osteosarcoma.**

| Biological interventions | Study phase | Clinical treatment effect | First posted time | ClinicalTrails.gov Identifier | Ref |
| --- | --- | --- | --- | --- | --- |
| Combination chemotherapy, PEG-interferon alfa-2b, and surgery | Phase 3 | Grade 3-4 neutropenia affected 83% of cycles and 59% were complicated by infection. There were three (0.13%) deaths related to pre-operative chemotherapy. | 24-Aug-05 | NCT00134030 | (1) |
| The anti-PD1 antibody pembrolizumab | Phase 2 | Pembrolizumab showed encouraging activity in patients with undifferentiated pleomorphic sarcoma or dedifferentiated liposarcoma. | 25-Nov-14 | NCT02301039 | (2) |
| Apatinib plus anti-PD1 therapy | Phase 2 | The 6-month progression-free survival of patients with advanced osteosarcoma was prolonged, but not reach 60%. | 2-Dec-17 | NCT03359018 | (3) |
| Camrelizumab in combination with neoadjuvant chemotherapy | Phase 2 | Clinical trials still in progress. | 4-Mar-20 | NCT04294511 | - |
| ZKAB001 5mg/kg  ZKAB001 10mg/kg  ZKAB001 15mg/kg | Phase 1  Phase 2 | Clinical trials still in progress. | 19-Sep-18 | NCT03676985 | - |

**References**

1. Whelan JS, Bielack SS, Marina N, Smeland S, Jovic G, Hook JM, et al. Euramos-1, an International Randomised Study for Osteosarcoma: Results from Pre-Randomisation Treatment. *Ann Oncol* (2015) 26(2):407-14. Epub 20141124. doi: 10.1093/annonc/mdu526.

2. Tawbi HA, Burgess M, Bolejack V, Van Tine BA, Schuetze SM, Hu J, et al. Pembrolizumab in Advanced Soft-Tissue Sarcoma and Bone Sarcoma (Sarc028): A Multicentre, Two-Cohort, Single-Arm, Open-Label, Phase 2 Trial. *Lancet Oncol* (2017) 18(11):1493-501. Epub 20171004. doi: 10.1016/S1470-2045(17)30624-1.

3. Xie L, Xu J, Sun X, Guo W, Gu J, Liu K, et al. Apatinib Plus Camrelizumab (Anti-Pd1 Therapy, Shr-1210) for Advanced Osteosarcoma (Apfao) Progressing after Chemotherapy: A Single-Arm, Open-Label, Phase 2 Trial. *J Immunother Cancer* (2020) 8(1). doi: 10.1136/jitc-2020-000798.
