# Supplementary figures and images for: Modulation of immunosuppressive cells and noncoding RNAs as immunotherapy in osteosarcoma
Source: Front Immunol. 2022 Nov 15;13:1025532. doi: 10.3389/fimmu.2022.1025532 (PMC9705758; doi:10.3389/fimmu.2022.1025532)

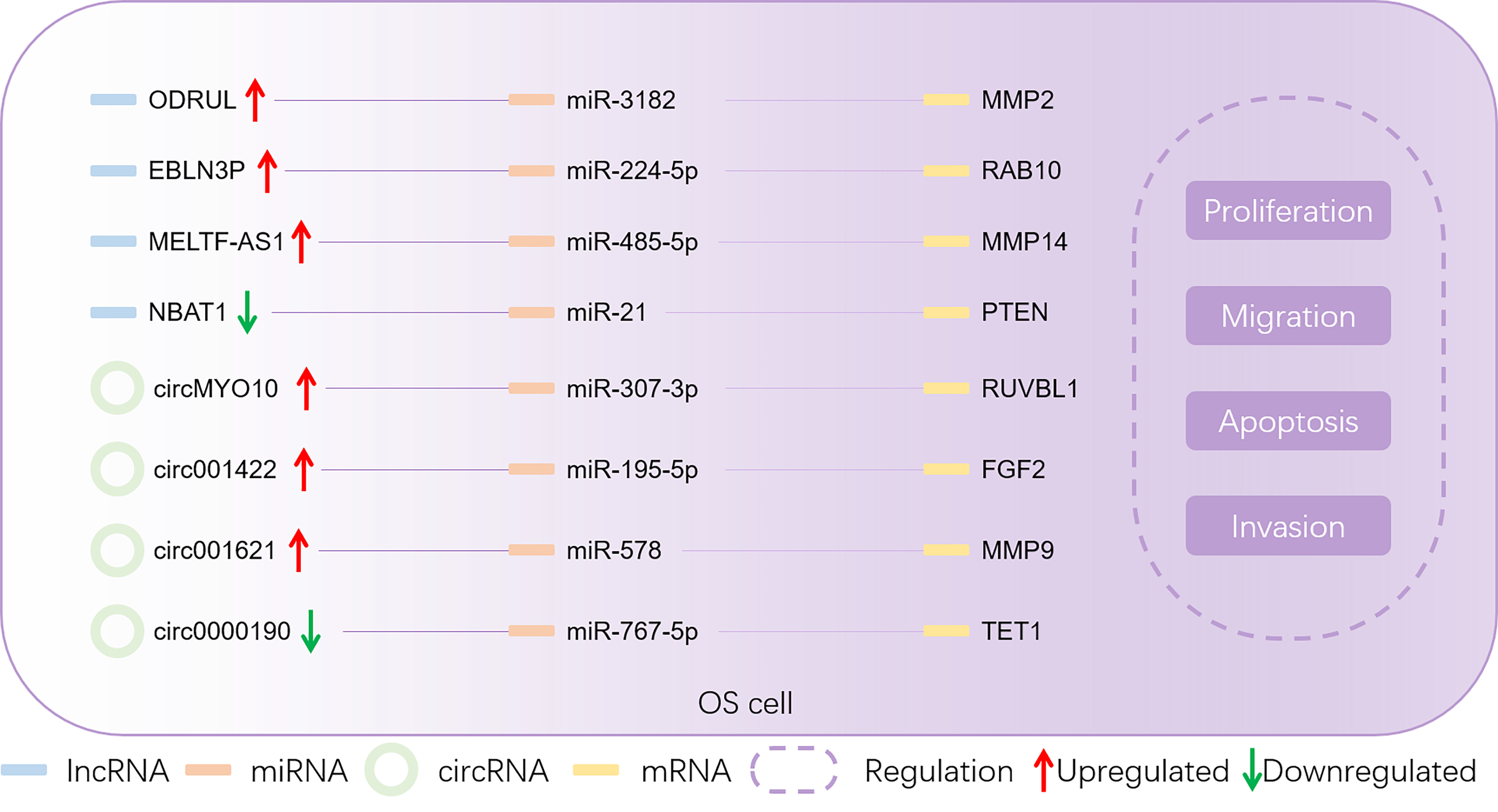

Supplement: Supplementary Figure 1 — The ceRNA network in OS cells. [file Image_1.tif]
